# Cognitive Interventions in Individuals With Chronic Respiratory Diseases: Protocol for a Systematic Review

**DOI:** 10.2196/48235

**Published:** 2023-07-28

**Authors:** Danielle Ryzer, Bushra Bhatti, Alana Streicher, Paula Weinberg, Fady Hanna, Jessica Moretto, Dina Brooks, Shirley Quach, Ana Oliveira

**Affiliations:** 1 School of Rehabilitation Science McMaster University Hamilton, ON Canada; 2 Respiratory Research West Park Healthcare Center Toronto, ON Canada; 3 Department of Medicine, Rehabilitation Science Institute and Physical Therapy University of Toronto Toronto, ON Canada; 4 Respiratory Research and Rehabilitation Laboratory School of Health Sciences, University of Aveiro Aveiro Portugal; 5 Institute of Biomedicine, Department of Medical Sciences University of Aveiro Aveiro Portugal

**Keywords:** chronic pulmonary disease, cognitive therapy, memory training, brain exercise, cognitive function, pulmonary, lung, COPD, review methodology, systematic review, cognitive, cognition, memory, CRD, respiratory

## Abstract

**Background:**

Chronic respiratory diseases (CRDs) may cause reduced oxygen availability to organs and body tissues, leading to an increased risk for ischemic damage, which can result in brain tissue injury. This damage can lead to a myriad of neurological symptoms contributing to cognitive decline. Cognitive interventions may attenuate cognitive deficits in people with CRDs; however, the effects have not yet been systematically summarized in the literature.

**Objective:**

The purpose of this systematic review is to assess the effects of cognitive interventions (including cognitive behavioral therapy and transcranial brain stimulation) on cognitive function (primary outcome), HRQL, self-management, symptoms, physical activity, physical function, ability to complete activities of daily living (ADLs), hospital admissions, functional capacity, functional performance, psychological and social outcomes, exacerbations, healthcare utilization, and survival in individuals with CRDs.

**Methods:**

This review will be conducted in accordance with the Cochrane handbook for systematic reviews of interventions and reported following the PRISMA (Preferred Reporting Items for Systematic Reviews and Meta-Analyses) guidelines. Searches will be performed in MEDLINE, Embase, Emcare, PsycINFO, Scopus, and CINAHL. Articles will be included if they focus on the effects of cognitive interventions on adults with CRDs, are published in peer-reviewed journals, and are written in English, French, or Portuguese. Risk of bias will be evaluated with the Cochrane Risk of Bias 2 tool for randomized controlled trials, and the Risk of Bias in Non-randomized Studies of Interventions tool for nonrandomized studies. Meta-analyses will be performed if at least 2 studies provided sufficient data for a specific outcome. The GRADE (Grading of Recommendations, Assessment, Development, and Evaluation) assessment will be used to evaluate the overall quality of the evidence.

**Results:**

This systematic review was initiated in November 2022 and registered with PROSPERO in February 2023, prior to title and abstract screening. Full-text screening of articles will be completed in June 2023. Data extraction and drafting of the manuscript will occur from July 2023 to August 2023, with expected publication in February 2024.

**Conclusions:**

This systematic review will summarize the effects of cognitive interventions on cognitive function in people with CRDs. It will guide health care professionals in selecting evidence-based strategies to enhance cognitive well-being and overall health outcomes for individuals with CRDs. Additionally, it will identify research gaps and highlight areas for future exploration, supporting researchers in advancing knowledge in this field.

**Trial Registration:**

PROSPERO CRD42023396234; https://tinyurl.com/mwjrfbxv

**International Registered Report Identifier (IRRID):**

PRR1-10.2196/48235

## Introduction

Chronic respiratory diseases (CRDs) are the leading causes of morbidity and mortality, contributing to three million deaths per year worldwide and US $170.8 billion in health care–related costs in 2016 [1]. Clinical presentation of CRDs includes both pulmonary and extrapulmonary symptoms, including dyspnea, cough, musculoskeletal impairments, and cognitive decline, ultimately limiting individuals’ abilities to engage in activities of daily living (ADLs) [2].

Cognitive decline is reported in 60% of people with CRDs and has been related to a state of reduced oxygen availability in tissues, resulting in an increased demand for oxygen to maintain homeostasis [3,4]. As CRDs progress, physiological changes such as airway obstruction, inflammation, fibrosis, septal thickening, interstitial edema, intra-alveolar exudate, and destruction of alveolar capillaries can reduce airflow and pulmonary gas diffusion. Such changes lead to impaired exchange of oxygen and carbon dioxide, resulting in hypoxemia, hypoxia, and hypercapnia [5,6]. If maintained, hypoxemia can affect the synthesis of neurotransmitters essential for brain function, such as acetylcholine [4], and cause ischemic brain damage [5,6].

Consistent cerebral hypoxia can present as a multitude of neurological symptoms associated with cognitive decline, including deficits related to memory, information processing, language, attention, visuospatial abilities, judgment, and executive functioning [3,7-9]. As a result, an individual’s ability to manage their disease, such as medication tracking and symptom monitoring, may be impeded by this cognitive decline [9,10]. Thus, maintaining and improving cognitive status is crucial to help promote disease self-management and independence in individuals with CRD.

Cognitive interventions encompass a range of targeted approaches designed to maintain or improve specific cognitive functions, such as attention, memory, and reasoning [11,12]. These interventions may involve structured training programs, therapeutic techniques, and innovative methods like transcranial brain stimulation (TBS). They are aimed at improving impaired cognitive function, preventing cognitive decline, or preserving the individual's functional level, drawing from diverse theoretical constructs [13]. Previous studies on non-CRD populations have reported improvements in general cognitive functioning, processing speed, and cognitive abilities related to everyday task performance following cognitive interventions [14]. In addition, these interventions have been reported to slow the progression of cognitive decline, particularly in populations with cognitive impairment [15]. However, the literature on the effects of cognitive interventions for people with CRDs is inconclusive, as it is heterogeneous in terms of sample characteristics, interventions, and outcome measures. Several studies, including randomized controlled trials (RCTs) investigating cognitive interventions in patients with chronic obstructive pulmonary disease, have produced inconsistent and variable findings [16-18]. Although there are possible benefits of cognitive interventions in those with CRDs, such as improvements in cognitive function, self-management, health-related quality of life (HRQL), and performance in trained tasks [16,17], overall, no significant improvement has been found related to cognitive performance, healthy lifestyle behaviors, susceptibility to cognitive stress and cognitive performance [16-18]. Consequently, a review is needed to systematically review and summarize the evidence, and establish its quality to help inform clinical practice as it pertains to the use of cognitive interventions in those with CRDs.

This systematic review primarily aims to assess the effects of cognitive interventions (ie, attention, memory, and reasoning on cognitive function) in people with CRDs. The secondary objectives are to summarize the content and structure of cognitive interventions used within this population and their effects on HRQL, self-management, symptoms, physical activity, physical function, ability to complete ADLs, hospital admissions, functional capacity, functional performance, psychological and social outcomes, exacerbations, healthcare utilization, and survival.

## Methods

### Overview

This systematic review will be conducted according to the Cochrane Handbook for Systematic Reviews of Interventions [19] and reported following the PRISMA (Preferred Reporting Items for Systematic Reviews and Meta-Analyses) guidelines [20]. The protocol was registered in PROSPERO (CRD42023396234).

### Search Strategy

The following electronic databases will be searched in June 2023: MEDLINE (1946-Present), Emcare, Embase, CINAHL, PsycINFO, and Scopus. The search strategy was created in consultation with a health sciences librarian at McMaster University and will involve database-specific MeSH terms in combination with keywords (keywords will be consistent across databases). The reference list of the selected studies will be manually searched to identify further relevant articles. The search strategy was validated in MEDLINE, Emcare, and Embase databases by testing whether it could identify 3 relevant articles to be included, previously sought through Google Scholar [16-18].

### Eligibility Criteria

#### Study Design

Studies will be included if they are RCTs, non-RCTs, pre-post study designs, cohort studies, and case-control studies (all published in peer-reviewed journals). Studies will be excluded if they have been published in conference proceedings, magazines, news articles, dissertations, theses, abstracts, and editorials. Systematic, scoping, literature, and narrative reviews will be excluded but their references will be screened for potential articles. Articles that are not written in English, French, or Portuguese will also be excluded.

#### Population

Studies will be included if they included participants 18 years of age or older (adult participants) with a diagnosis of a CRD (ie, asthma, bronchiectasis, chronic obstructive pulmonary disease, cystic fibrosis, interstitial lung disease, chronic respiratory tract diseases, post–COVID-19 syndrome, tuberculosis, or lung cancer). Studies will be excluded if they include participants whose primary diagnosis is a cognitive disease or participants presenting with complications such as dementia, Alzheimer, psychological disorders (ie, bipolar disorder), stroke, or trauma, resulting in cognitive impairment.

#### Intervention

Studies will be included if reporting on the effects of cognitive interventions. Cognitive interventions that help improve or maintain cognitive processes or address the impact of cognitive impairment on overall function will be included. These interventions may involve formally structured training in domains such as information processing speed, attention, memory, or problem-solving. The focus should be on improving the reasoning or cognitive process itself rather than developing a specific skill [21]. As per our definitions, cognitive training and cognitive interventions that include cognitive behavioral therapy (CBT) or TBS focused on improving cognition will be included [22-24]. Cognitive training can be defined as nonpharmacological interventions that involve structured and guided training that aims to maintain or improve certain aspects of cognitive function, including attention, memory, and learning [21]. CBT is defined as an intervention that involves increasing participants' knowledge and understanding of the problem (ie, cognitive function, including attention, memory, and learning); identifying and restructuring dysfunctional thinking and maladaptive beliefs; and developing emotional and behavioral compensatory strategies for the core deficits [22]. It is a diverse group of treatments that include cognitive therapy, CBT, acceptance and commitment therapy, dialectical behavior therapy, schema-focused therapy, rational-emotive behavior therapy, mindfulness-based cognitive therapy, metacognitive therapy, cognitive behavioral analysis system of psychotherapy, and cognitive processing therapy [23]. Transcranial brain stimulation is a non-invasive brain stimulation technique that passes an electrical current through the cortex of the brain to alter brain function [24]. It may include transcranial direct current stimulation, alternating current stimulation, and random noise stimulation [24].

Studies that primarily focus on improving and changing behavioral, emotional, or physical outcomes, single-trial training, and interventions implemented in direct combination with another distinct experimental intervention will be excluded [21].

Having a comparator or control group is not a requirement for inclusion in this review. Accepted types of control groups will be no intervention, usual care, sham intervention, another type of intervention, or multidimensional care (cognitive intervention plus another intervention). Control groups involving another type of cognitive intervention will not be accepted, as this will only determine the effectiveness of one intervention versus the other and not whether cognitive interventions are effective compared to no intervention or another type of intervention.

#### Outcomes

The primary outcome of this study is cognitive function, which encompasses the mental processes involved in acquiring knowledge, manipulating information, and reasoning [25]. This includes the domains of perception, memory, learning, attention, decision-making, and language abilities [25]. Secondary outcomes include HRQL, self-management, symptoms, physical activity, physical function, ability to complete ADL, hospital admissions, functional capacity, functional performance, psychological and social outcomes, exacerbations, health care usage, and survival.

If sufficient data are available from 2 or more studies, subgroup analyses will be performed for the type of CRD and the structure (ie, duration, frequency, and setting) or components of the cognitive intervention. Such analysis will help determine whether patients with different CRDs respond differently to cognitive interventions, or whether there is a difference in outcomes depending on the cognitive intervention structure or components.

### Data Management

EndNote reference manager (Clarivate) will be used to filter duplicate studies. Studies will subsequently be imported into Covidence (Veritas Health Innovation), which will be used for further removal of duplicates, screening, and selecting studies. Excel (Microsoft Corp) will be used for data extraction and management.

### Selection Process

Abstracts and full-text screening will be pilot-tested by all reviewers with 12 articles to calibrate the selection process and ensure interrater reliability. An 80% or higher agreement will be sought before proceeding with screening [26]. Six reviewers will independently screen titles and abstracts, with each study requiring consensus between 2 reviewers before a final decision to include or exclude the study for the full-text screening is made [26]. Two authors will independently perform the full-text screening process. At any stage of the screening process, if articles do not meet the inclusion criteria or meet any of the exclusion criteria, they will be excluded and reasons for exclusion will be recorded [26]. Disagreements will be resolved through consensus, and if no consensus is reached, a third reviewer will be consulted.

### Data Extraction

Two authors will independently extract the data to a predefined data extraction table, including title, authors, year and country of publication, study design, study setting, sample size per group, population (age, gender, chronic respiratory disease, disease severity, and comorbidities) per group, intervention (type, frequency, components, and duration) per group, outcome measures used, the timing of data collection, follow-up times if applicable, adverse events, and results of the interventions or comparator groups (including the mean, SD, and sample size).

Any disagreements on the extracted data will be resolved by discussion, and a third reviewer will be consulted if no consensus is reached. In the case there are missing data, study authors will be contacted by email to request these data. A follow-up request will be sent after 2 weeks, and if there continues to be no response after 2 more weeks, there will be no further attempts to retrieve the data. If the data are reported only in figures and the authors did not provide the requested data, whenever possible WebPlotDigitizer will be used [27].

### Risk of Bias Assessment

Risk of bias assessment will be performed independently by 2 authors at a study level using the Cochrane Risk of Bias 2 tool for RCTs (randomization process, deviations from intended interventions, missing outcome data, measurement of the outcome, and selection of the reported result) or the Risk of Bias in Non-randomized Studies of Interventions tool for non-RCTs (bias due to confounding, bias of selection of participants into the study, bias in classification of interventions, bias due to deviations from intended interventions, bias due to missing data, bias in measurement of outcomes, and bias in selection of the reported result) [28,29]. Disagreements will be solved through discussion, and if no consensus is reached, a third reviewer will be consulted.

### Data Analysis and Synthesis

Tables will be used to describe the various types of studies, patient populations, characteristics of cognitive interventions and comparators, and outcomes. Meta-analysis will be performed using the Review Manager (version 5; Cochrane) software. If at least 2 studies reported the same outcome, that outcome will be included in the meta-analysis. Results will be reported as mean difference if the studies used the same outcome measures or standardized mean difference if studies assessed the same outcome using different measures. Depending on the level of heterogeneity between applicable studies, a fixed effects or a random effects model will be used for meta-analysis (ie, a high level of heterogeneity between applicable studies would warrant the use of a random effects model). If a meta-analysis cannot be performed, data will be synthesized narratively, explaining the main findings as they relate to the impact of cognitive interventions for people with CRDs.

### Quality of Evidence

For the quality of evidence assessment, the GRADE (Grading of Recommendations, Assessment, Development, and Evaluation) system will be used to upgrade or downgrade the evidence [30]. The GRADE assessment will be performed independently by 2 authors and disagreements will be solved through consensus or consulting a third reviewer if a consensus cannot be reached. The domains to be looked at are as follows: risk of bias, imprecision, inconsistency, indirectness, and publication bias. Based on these domains, the quality of evidence will be determined to be high, moderate, low, or very low. The outcome of the GRADE assessment will be presented in a summary of the findings table.

## Results

The project was initiated in November 2022. After formalizing the study methods and prior to title and abstract screening, the protocol was registered with PROSPERO in February 2023. Abstract screening is planned to be completed in June 2023 and full-text screening will be completed in July 2023. Data analysis and drafting of the manuscript will occur from July 2023 to August 2023, with expected publication in February 2024.

## Discussion

### Principal Findings

The beneficial effects of cognitive interventions for healthy individuals and those with various cognitive impairments have been discussed in several studies [14-17]. However, its effects on people with CRDs have never been systematically documented [4,16-18]. To our knowledge, this is the first systematic review focusing on the effects of cognitive intervention in individuals with CRDs. The nature of this systematic review is a strength, as it provides an objective perspective on this topic while minimizing bias, by following established procedures and reporting guidelines. Other strengths include its robust search strategy as demonstrated by searching 6 databases, reporting on the percentage of agreement among reviewers and including a variety of health domains in the secondary outcomes.

One of the predicted limitations of this study is that it will collect data from various study types, not limited to RCTs. This may affect the quality of the literature included in the paper. However, due to the novelty of this rehabilitation topic, there may be a limited number of primary articles (RCTs) available. Despite this limitation, it is still essential to synthesize the current literature on the effects of cognitive interventions for people with CRDs to inform evidence-based clinical decisions and plan future research. Another limitation of the study is the inclusion of articles written only in English, Portuguese, and French. This may exclude relevant data published in other languages. To capture a broader range of articles, future research on this topic should include studies written in other languages. Finally, to ensure a focused and manageable analysis, we have made the decision to include only the most commonly reported forms of cognitive interventions in the literature. This selective approach allows us to streamline our review process and maintain clarity in our findings. Although this decision may exclude certain less frequently reported interventions, it enables us to provide a comprehensive evaluation of the widely recognized and established cognitive intervention techniques.

### Conclusions

The systematic review's findings will assist in displaying the effectiveness of cognitive interventions as a potential treatment approach in reducing cognitive deficits in those with CRDs. This innovative field of clinical research is relevant to all health care disciplines as it impacts decisions related to screening and interventions and informs clinical practice for all health care professionals.

## Abbreviations

ADL: activity of daily living
CBT: cognitive behavioral therapy
CRD: chronic respiratory disease
GRADE: Grading of Recommendations, Assessment, Development, and Evaluation
HRQL: health-related quality of life
PRISMA: Preferred Reporting Items for Systematic Reviews and Meta-Analyses
RCT: randomized controlled trial
TBS: transcranial brain stimulation

## Data Availability

All data generated or analyzed during this study will be included in the published manuscript as a supplementary information file.
